# Prediction of Long‐Term Prognosis in Patients With Hepatocellular Carcinoma Using the National Clinical Database Risk Calculator

**DOI:** 10.1002/ags3.70256

**Published:** 2026-07-26

**Authors:** Mariko Tsukagoshi, Kenichiro Araki, Norio Kubo, Takamichi Igarashi, Shunsuke Kawai, Kei Hagiwara, Kouki Hoshino, Ryo Muranushi, Ken Shirabe

**Affiliations:** ^1^ Division of Hepatobiliary and Pancreatic Surgery, Department of General Surgical Science Gunma University Graduate School of Medicine Maebashi Gunma Japan

**Keywords:** hepatocellular carcinoma, National Clinical Database, prognosis, risk calculator, surgery

## Abstract

**Aim:**

To determine the value of the National Clinical Database (NCD) risk calculator for predicting surgical outcomes and long‐term prognosis in patients undergoing resection for hepatocellular carcinoma (HCC).

**Methods:**

We retrospectively analyzed data from 210 patients with HCC who underwent initial hepatic resection between January 2016 and December 2022. We calculated predicted incidence of surgery‐related mortality (predicted mortality rate) using the NCD risk calculator. We assessed the relationship between predicted mortality rate and clinicopathological factors, including their relationship with long‐term prognosis.

**Results:**

A predicted mortality rate of ≥ 3% significantly correlated with worse recurrence‐free survival and overall survival (OS), with a high‐risk group showing increased blood loss, longer hospital stays, and higher severe complication rates. Multivariate analysis identified predicted mortality rate of ≥ 3% as an independent prognostic indicator for poor OS, alongside male sex and microvascular invasion.

**Conclusion:**

The NCD risk calculator showed potential to predict not only short‐term outcomes but also long‐term prognosis in patients with HCC undergoing resection. The NCD risk calculator may be a valuable complementary tool for decision‐making about liver resection in patients with HCC.

## Graphical abstract

This study aimed to determine the value of the National Clinical Database (NCD) risk calculator in predicting surgical outcomes and long‐term prognosis in patients undergoing resection for hepatocellular carcinoma (HCC). We retrospectively analyzed data from 210 patients with HCC who underwent initial hepatic resection, assessing the relationship between the predicted mortality rate using the NCD risk calculator and various clinicopathological factors. A predicted mortality rate of ≥ 3% significantly correlated with worse prognosis and higher complication rates, demonstrating that the NCD risk calculator is a valuable complementary tool for long‐term prognostic prediction and surgical decision‐making.

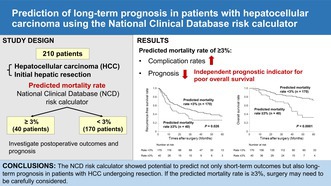

## 1 Introduction

Hepatocellular carcinoma (HCC) is the sixth most common cancer worldwide, and the third leading cause of cancer mortality [1]. Liver resection is one of the main curative treatments for HCC. However, patients with HCC have a high rate of recurrence, which remains the major cause of death, even after curative resection [2]. The 5‐year overall survival (OS) rate after liver resection is 46%–56%, and the 5‐year recurrence‐free survival (RFS) rate is 23%–32% [3, 4, 5]. To improve the long‐term prognosis of patients with HCC, it is necessary to identify patients at a high risk of recurrence early, determine whether liver resection is appropriate, and carefully consider other treatment options.

The National Clinical Database (NCD), a general incorporated association, is the largest and best organized nationwide surgical registry in Japan. Data entry began in 2011, and currently, more than 5400 facilities are participating, registering information on surgery and treatment, as well as patient information, to verify the effectiveness and risks of treatment [6]. More than 95% of surgeries in this area performed in Japan are registered, making the data extremely reliable [7]. One of the findings published in clinical studies using NCD data is feedback implementation (risk calculator) for quality improvement in cancer treatment [8]. The risk calculator for nine gastrointestinal procedures, including hepatectomy, is available and provides the predicted mortality of patients simultaneously with preoperative variable inputs.

The NCD calculator is useful in predicting short‐term surgical outcomes. By obtaining objective preoperative data on the risks patients face from surgery, we can determine treatment indications and obtain informed consent based on the results. However, there is no data on long‐term prognosis in NCDs, and the usefulness of the NCD risk calculator for long‐term prognosis has not yet been studied.

Therefore, we aimed to determine the value of the NCD risk calculator in predicting surgical outcomes and long‐term prognosis in patients undergoing liver resection for HCC.

## 2 Methods

### 2.1 Study Design and Participants

We performed a retrospective study of 210 patients with HCC who underwent initial hepatic resection between January 2016 and December 2022 at the Department of Hepatobiliary and Pancreatic Surgery of Gunma University Hospital. This study was approved by the Ethics Committee of the hospital (IRB2024‐044 (2226)), and institutional guidelines of the Declaration of Helsinki were met.

### 2.2 Data Collection and Treatment

Demographic and clinicopathological characteristics and treatment‐related details of all the patients were collected from their medical records. Surgical procedures were performed according to institutional policies and cancer board recommendations. Postoperative complications within 30 days were recorded and scored according to the Clavien–Dindo classification [9]. Resected tumors were classified according to the TNM Classification of Malignant Tumors of the Union for International Cancer Control (8th edition).

### 2.3 NCD Risk Calculator

Predicted incidence of surgery‐related mortality (predicted mortality rate) was calculated using the NCD risk calculator (https://www.ncd.or.jp/about/feedback.html). Preoperative risks used in the calculator included the following: age, sex, ambulance transport, emergency surgery, height, weight, body mass index, diabetes, smoking, difficulty breathing, activities of daily living, mechanical ventilation management, chronic obstructive pulmonary disease, pneumonia, ascites, high blood pressure, cardiac surgery, peripheral vascular disease surgery, dialysis, cerebrovascular disease, multiple metastases, open wounds, long‐term steroid administration, weight loss, blood coagulation disorders, blood transfusions, sepsis, white blood cells, hemoglobin, hematocrit, platelets, albumin, total bilirubin, aspartate aminotransferase, alkaline phosphatase, urea nitrogen, creatinine, serum sodium, C‐reactive protein (CRP), prothrombin time, prothrombin time international normalized ratio, and surgery information.

Predicted mortality rate of the NCD risk calculator was calculated for all the patients before surgery, which was used to determine whether surgery was appropriate. In our hospital, patients with ≥ 10% predicted mortality rate were generally not considered candidates for surgery. For patients with 5%–10% predicted mortality rate, if alternative treatments were difficult, surgery was considered appropriate after necessary preoperative interventions were performed, and the patients and their families were informed of the high risk.

### 2.4 Follow‐Up

All the patients were examined every 3 months for recurrence after discharge using tumor markers and computed tomography (CT) or magnetic resonance imaging. OS was defined as the period from the date of surgery to the date of all‐cause mortality. RFS was defined as the period from the date of surgery to the date of documented recurrence or all‐cause mortality. Recurrent HCC was treated with surgery, radiotherapy, chemotherapy, transcatheter arterial chemoembolization, or heavy‐ion radiotherapy, depending on the recurrence status.

### 2.5 Statistical Analysis

Categorical variables were assessed using the chi‐squared or Fisher's exact test, as appropriate. Continuous variables were summarized as median values (with interquartile ranges) and compared using the Mann–Whitney *U* test. Survival curves were estimated using the Kaplan–Meier method, and the log‐rank test was used to analyze differences between curves. Cox proportional hazards model analysis was performed using univariate and multivariate analyses of prognostic factors. All statistical analyses were performed using JMP Pro 18 software (SAS Institute, Cary, NC, USA). A *p* < 0.05 was considered statistically significant.

## 3 Results

### 3.1 Clinical Characteristics of Patients According to Predicted Mortality Rate

**FIGURE 1:**
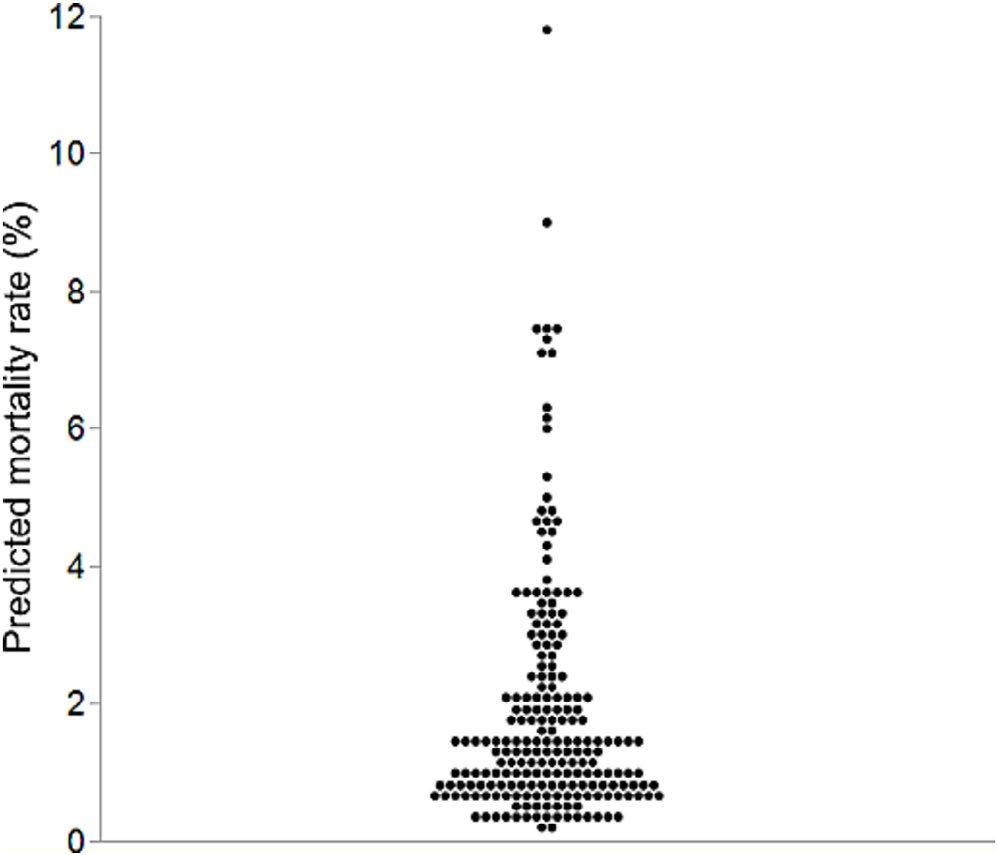
Distribution of predicted mortality rates for all patients. Median predicted mortality rate was 1.3% (0.2%–11.8%).

A total of 210 patients were included in this study. Distribution of predicted mortality rates for all the patients is shown in Figure 1. Median predicted mortality rate was 1.3% (0.2%–11.8%). In one case, surgery was performed despite a predicted mortality rate exceeding 10%. In this case, the predicted 30‐day postoperative mortality rate was in the 6% range. The patient was over 85 years old, had a low platelet count due to hepatitis C, and exhibited mild renal impairment, which was thought to have contributed to the high predicted mortality rate. Since the patient's liver function and nutritional status were good and the lesion was a solitary HCC that was partially resectable, surgery was performed, and the postoperative course was uneventful.

Because a predicted mortality rate of ≥ 3% is considered a moderate surgical risk, we set 3% as the cut‐off value in this study. Patient characteristics are summarized in Table 1. Among them, 40 (19.0%) patients had a predicted mortality rate of ≥ 3%, while 170 (81.0%) had a rate of < 3%. Patients with predicted mortality rate of ≥ 3% were significantly older than those with < 3% (70 vs. 77 years, *p* < 0.0001). Platelet count, lymphocyte count, and albumin level were significantly lower, and CRP was significantly higher in patients with a predicted mortality rate of ≥ 3%. In patients with a predicted mortality rate of ≥ 3%, indocyanine green retention rate at 15 min (ICG–R15%) was significantly higher and Child–Pugh Score A was significantly lower. No significant differences were observed in sex, skeletal muscle mass index, or tumor marker levels. Regarding surgery‐related factors, patients with a predicted mortality rate of ≥ 3% had significantly higher blood loss (239 vs. 126 mL, *p* = 0.002), longer postoperative hospitalization (14 vs. 11 days, *p* = 0.001), and a higher rate of Clavien–Dindo grade ≥ 3 complications (28 vs. 11%, *p* = 0.012). There was no significant difference in tumor‐related factors between the two groups. A total of 28 (70%, predicted mortality rate of ≥ 3% group) and 90 (53%, predicted mortality rate of < 3% group) patients experienced HCC recurrence. There was no significant difference in the recurrence patterns between the two groups.

**TABLE 1:** Patient characteristics.

Variables	Predicted mortality rate < 3%	Predicted mortality rate ≥ 3%	
	(*n* = 170)	(*n* = 40)	*p*
Host‐related factors			
Age (years)	70 (18–90)	77 (62–87)	< 0.0001*
Sex: Male	139 (82%)	33 (83%)	1.000
BMI (kg/m^2^)	23.1 (17.0–34.3)	22.8 (19.1–30.0)	0.732
Skeletal muscle mass index (cm^2^/m^2^)	39.4 (20.7–72.2)	35.9 (28.1–50.2)	0.067
Hand grip strength (kg)	33.0 (8.6–49.9)	27.4 (16.7–40.7)	0.050
Etiology HBV/HCV/NBNC	22/65/77	0/13/26	0.019*
Platelet count (/μL)	17.1 (5.6–57.7)	13.3 (6.3–30.3)	0.001*
Lymphocytes (/μL)	1525 (340–3410)	1175 (670–2720)	0.015*
PT (%)	93 (11–121)	94 (46–116)	0.558
Total bilirubin (mg/dL)	0.8 (0.2–3.1)	0.9 (0.3–2.0)	0.760
Albumin (mg/dL)	4.2 (2.9–5.3)	3.9 (2.7–4.6)	< 0.0001*
AST	31 (12–186)	38 (14–192)	0.044*
ALT	26 (7–167)	32 (9–272)	0.114
CRP (mg/dL)	0.09 (0.01–8.93)	0.21 (0.02–8.37)	0.036*
ICG‐R15 (%)	14.1 (1.6–91.8)	17.7 (1.5–52.3)	0.003*
Child–Pugh Score A	167 (98%)	36 (90%)	0.026*
AFP (ng/mL)	10.2 (1.0–108 317)	12.6 (1.0–275 819)	0.681
Surgery‐related factors			
Anatomical resection	95 (56%)	25 (63%)	0.482
Minimally invasive surgery	61 (36%)	9 (23%)	0.136
Operation time (min)	339 (105–643)	356 (150–682)	0.613
Blood loss (mL)	126 (0–2050)	239 (0–7219)	0.002*
Postoperative hospitalization (days)	11 (5–141)	14 (7–196)	0.001*
Complications (Clavien–Dindo grade ≥ 3)	19 (11%)	11 (28%)	0.012*
Tumor‐related factors			
Tumor size (mm)	3.1 (0.7–22.0)	4.1 (01.2–16.0)	0.091
Multiple tumors	32 (19%)	8 (20%)	0.826
Poor differentiation	30 (18%)	10 (25%)	0.370
Microvascular invasion (+)	65 (38%)	21 (53%)	0.110
Recurrence	90 (53%)	28 (70%)	0.054
Extrahepatic recurrence	18 (11%)	9 (23%)	0.063
Early (within 1 year after surgery) recurrence	53 (31%)	19 (48%)	0.064

*Note:* Data are expressed as median (interquartile range), or number of patient (%).

Abbreviations: AFP, alpha‐fetoprotein; ALT, alanine aminotransferase; AST, aspartate aminotransferase; BMI, body mass index; CRP, C‐reactive protein; HBV, hepatitis B virus; HCV, hepatitis C virus; ICGR‐15, indocyanine green retention rate at 15 min; NBNC, Non‐B, Non‐C Hepatocellular Carcinoma; PT, prothrombin time.

* *p* < 0.05.

### 3.2 Analysis of Long‐Term Postoperative Prognosis by Predicted Mortality Rate

**FIGURE 2:**
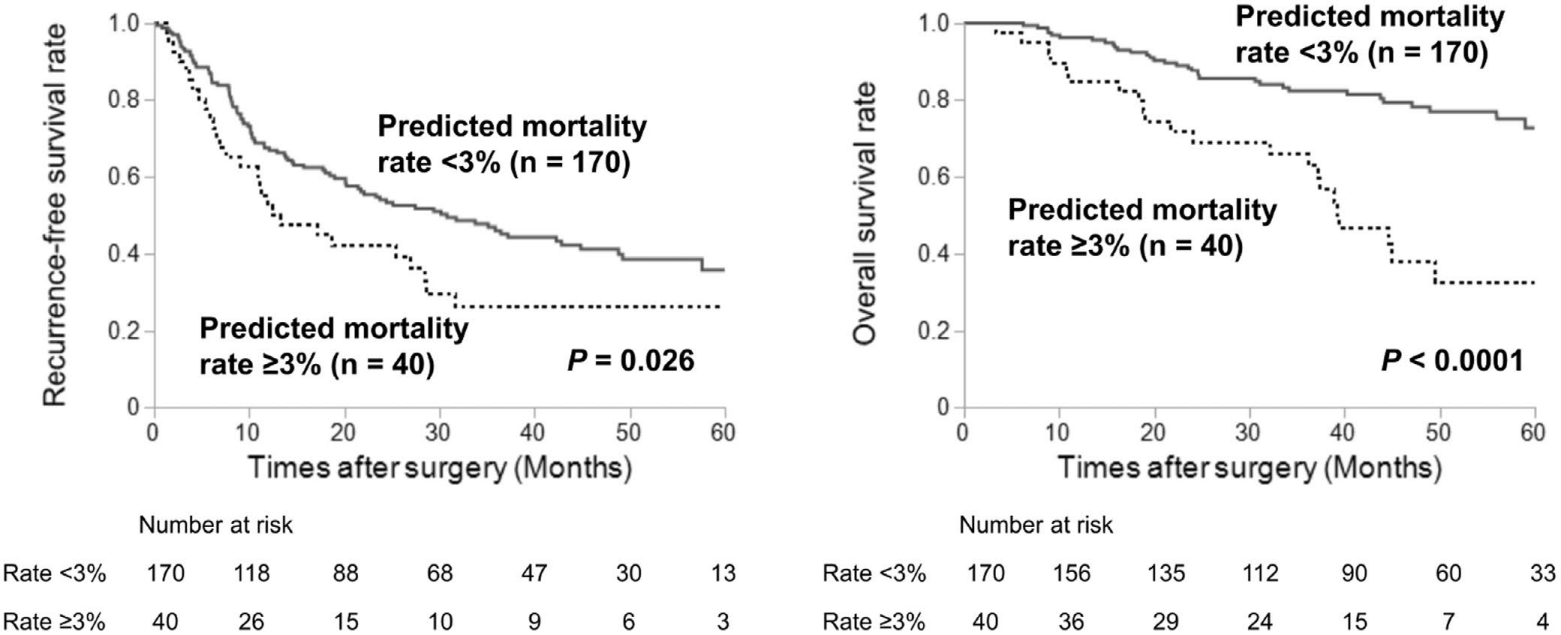
Postoperative long‐term prognosis analysis according to predicted mortality rate. Patients with predicted mortality rate ≥ 3% had significantly poorer recurrence‐free survival (RFS) than those with rates < 3% (*p* = 0.026). Median RFS time was 12.4 and 30.4 months in patients with predicted mortality rates of ≥ 3% and < 3%, respectively. Patients with predicted mortality rates of ≥ 3% had significantly poorer overall survival than those with rates of < 3% (median survival time: 39.2 months vs. not reached, *p* < 0.0001).

Figure 2 shows long‐term postoperative prognosis based on predicted mortality rate. Patients with predicted mortality rate of ≥ 3% had significantly poorer RFS than those with < 3% (*p* = 0.026). Median RFS time was 12.4 and 30.4 months in patients with predicted mortality rate of ≥ 3% and < 3%, respectively. Regarding the OS rate, patients with predicted mortality rate of ≥ 3% had significantly poorer OS than those with < 3% (median survival time: 39.2 months vs. not reached, *p* < 0.0001).

### 3.3 Prognostic Factors Associated With RFS and OS

Univariate and multivariate analyses were performed to analyze the factors influencing RFS in all the patients (Table 2). Univariate analysis revealed that being male, prognostic nutritional index (PNI) of < 45, geriatric nutritional risk index (GNRI) of < 98, blood loss of > 500 mL, tumor size of > 30 mm, microvascular invasion (MVI), and predicted mortality rate of ≥ 3% were significant factors for reduced RFS. Multivariate analysis revealed that male sex, blood loss of > 500 mL, and MVI were independent prognostic indicators of poor RFS.

**TABLE 2:** Univariate and multivariate analyses for recurrence‐free survival.

Variables	Univariate analysis			Multivariate analysis		
	HR	95% CI	*p*	HR	95% CI	*p*
Age > 80 (years)	0.86	0.50–1.49	0.604			
Sex: male	2.42	1.36–4.32	0.003*	2.85	1.59–5.12	0.0005*
Child–Pugh Score B or C	0.62	0.15–2.53	0.51			
PNI < 45	1.64	1.10–2.44	0.016*	1.22	0.74–2.02	0.428
GNRI < 98	1.61	1.04–2.52	0.034*	0.95	0.54–1.86	0.854
AFP > 40 (ng/mL)	1.16	0.79–1.70	0.439			
ICG‐R15 > 10 (%)	1.37	0.87–2.15	0.180			
Predicted mortality rate ≥ 3%	1.55	1.02–2.38	0.041*	1.30	0.84–2.02	0.243
Operation time > 300 (min)	1.33	0.91–1.95	0.144			
Blood loss > 500 (mL)	2.06	1.29–3.28	0.002*	1.68	1.02–2.77	0.041*
Complications (Clavien–Dindo grade ≥ 3)	1.32	0.82–2.13	0.260			
Tumor size > 30 (mm)	1.54	1.07–2.21	0.021*	1.32	0.90–1.94	0.160
Multiple tumors	1.39	0.89–2.16	0.142			
Poor differentiation	1.36	0.88–2.12	0.168			
Microvascular invasion (+)	2.35	1.63–3.37	< 0.0001*	2.20	1.51–3.19	< 0.0001*

Abbreviations: AFP, alpha‐fetoprotein; CI, confidence interval; GNRI, Geriatric Nutritional Risk Index; HR, hazard ratio; ICGR‐15, indocyanine green retention rate at 15 min; PNI, Prognostic Nutritional Index.

* *p* < 0.05.

Table 3 shows results of the univariate and multivariate analyses for predicting OS. A univariate analysis revealed that being a male, PNI of < 45, GNRI of < 98, operative time of > 300 min, blood loss of > 500 mL, complication (Clavien–Dindo grade ≥ 3), tumor size of > 30 mm, multi tumors, MVI, and predicted mortality rate of ≥ 3% as significant factors for reduced OS. The independent predictive factor detected by the multivariate analysis was male, MVI, and predicted mortality rate of ≥ 3% (Hazard ratio = 2.57; 95% confidence interval: 1.39–4.76; *p =* 0.003).

**TABLE 3:** Univariate and multivariate analyses for overall survival.

Variables	Univariate analysis			Multivariate analysis		
	HR	95% CI	*p*	HR	95% CI	*p*
Age > 80 (years)	1.76	0.91–3.42	0.094			
Sex: male	3.51	1.27–9.69	0.016*	4.82	1.69–13.71	0.003*
Child–Pugh Score B or C	1.85	0.45–7.62	0.394			
PNI < 45	2.23	1.30–3.83	0.004*	1.12	0.55–2.30	0.757
GNRI < 98	3.28	1.88–5.74	< 0.0001*	1.39	0.61–3.14	0.435
AFP > 40 (ng/mL)	1.55	0.91–2.64	0.103			
ICG‐R15 > 10 (%)	2.15	0.97–4.75	0.058			
Predicted mortality rate ≥ 3%	3.48	2.04–5.93	< 0.0001*	2.57	1.39–4.76	0.003*
Operation time > 300 (min)	2.33	1.21–4.50	0.012*	1.24	0.59–2.60	0.576
Blood loss > 500 (mL)	2.22	1.21–4.07	0.010*	1.72	0.88–3.38	0.112
Complications (Clavien–Dindo grade ≥ 3)	1.95	1.06–3.56	0.031*	1.05	0.53–2.08	0.894
Tumor size > 30 (mm)	2.06	1.19–3.58	0.010*	1.47	0.81–2.68	0.209
Multiple tumors	1.85	1.02–3.34	0.042*	1.51	0.80–2.85	0.203
Poor differentiation	1.67	0.89–3.10	0.108			
Microvascular invasion (+)	3.44	1.99–5.95	< 0.0001*	2.71	1.47–4.98	0.001*

Abbreviations: AFP, alpha‐fetoprotein; CI, confidence interval; GNRI, Geriatric Nutritional Risk Index; HR, hazard ratio; ICGR‐15, indocyanine green retention rate at 15 min; PNI, Prognostic Nutritional Index.

* *p* < 0.05.

## 4 Discussion

In this study, we identified the NCD risk calculator value for predicting surgical outcomes and long‐term prognosis in patients undergoing liver resection for HCC. A predicted mortality rate of ≥ 3% was significantly associated with increased postoperative complications and prolonged length of postoperative hospitalization. Furthermore, a predicted mortality rate of ≥ 3% was significantly associated with poorer RFS and OS compared with rates < 3%. A predicted mortality rate of ≥ 3% was an independent postoperative prognostic factor for OS.

The Japanese NCD is the largest nationwide database, which covers > 95% of the surgeries performed in Japan [8]. The NCD feedback systems were constructed and operated online based on risk models [10]. The accuracy of the NCD data was ensured by systematic audits for standard NCD input initiated in 2016 by the Japanese Society of Gastroenterological Surgery database committee [11]. For individual patients, a risk calculator can be used to estimate the probabilities of 30‐day mortality, operative mortality, and morbidity based on preoperative clinical information. The ability to determine individual surgical risks and predict mortality rates for patients preoperatively based on a large‐scale database is considered useful for physicians and patients when deciding on surgical indications. However, there is no defined threshold for what percentage constitutes a risk, leaving the decision to individual physicians.

In this study, we divided the patients into two groups using a 3% cut‐off value for predicted mortality rate. At our institution, patients with a ≥ 10% predicted mortality rate were generally not considered candidates for surgery. In comparison, patients with a 5%–10% predicted mortality rate were considered high‐risk, and alternative treatments and preoperative interventions were performed. Given that the surgery‐related mortality rate was 1.7% in an analysis using NCD data from 2014 to 2019 [12], a predicted mortality rate of 3% was considered a reasonable cut‐off for moderate surgical risk. Patients with predicted mortality rate of ≥ 3% were older and presented with impaired liver function (higher ICG‐R15% and lower Child–Pugh scores) and poor nutritional status (lower albumin and lower lymphocyte counts). These baseline factors in the patient background likely contributed to the significantly higher blood loss and increased rate of severe postoperative complications. These results demonstrate that predicted mortality rate according to the NCD risk calculator was useful for short‐term surgical outcomes, and the 3% cut‐off value we set was considered appropriate for evaluating short‐term surgical outcomes in patients with HCC.

The usefulness of the NCD risk calculator in predicting postoperative outcomes other than liver resection has been validated. Motono et al. [13] investigated 585 patients who underwent pulmonary resection for non‐small cell lung cancer, and risk factors for postoperative morbidity were analyzed to verify the validity of the NCD risk calculator. They compared risk score (mortality or severe morbidity) according to the risk calculator and actual postoperative morbidity by lobes and concluded that the NCD risk calculator for postoperative morbidity needs to be modified according to high‐risk lobes. Kimura et al. [14] examined older adult patients who underwent pancreaticoduodenectomy, predicted the incidence of decline in postoperative activities of daily living, and predicted that the incidence of Clavien–Dindo grade ≥ IV in the NCD risk calculator was a useful predictor of a poor postoperative course. Recently, Sugiyama et al. [15] examined whether frailty in older adults undergoing colorectal cancer surgery correlated with perioperative complication risks predicted by the NCD risk calculator. They showed that patients with a predicted fall risk of ≥ 52% had significantly higher rates of receiving palliative treatment, suffering postoperative complications, and not being discharged home. Because outcomes calculated using the NCD risk calculator differ vary by surgical procedure, it is difficult to directly apply results of other surgical procedures. There have been no previous reports examining the usefulness of the NCD risk calculator for liver resection or HCC, making this study the first of such report.

Furthermore, this study examined not only the original purpose of the NCD risk calculator in predicting postoperative complications and mortality but also its significance in long‐term prognosis. The long‐term prognostic factors for HCC after surgery are generally categorized into three groups: tumor‐related factors, liver factors, and perioperative patient indicators. In this study, no differences in tumor‐related factors were found between the two groups, with a 3% predicted mortality rate cut‐off. However, tumor‐related factors are important prognostic factors, and the presence of MVI is a well‐known poor prognostic factor. In fact, in this study, MVI was an independent prognostic factor for OS, along with male sex and predicted mortality rate of ≥ 3%. Therefore, a comprehensive risk assessment for long‐term prognosis requires a balanced consideration of both host‐related factors and tumor biology.

Previous studies have shown that MVI predicts worse prognosis after liver resection in patients with HCC [16, 17, 18]. However, MVI is diagnosed via histological examination of surgical specimens after hepatic resection, which limits its influence on preoperative decision‐making. Therefore, several studies have been conducted to predict the presence of MVI before liver resection [17, 19]. Based on these studies, preoperative factors associated with MVI were identified, including tumor diameter, multiple nodules, tumor markers, and platelet count. Recently, a study has also been conducted on deep learning‐based multi‐instance learning models for preoperative MVI prediction. Cen et al. [20] demonstrated that a multi‐instance learning model based on CT arterial phase images could effectively predict the MVI status of HCC and prognosis risk across different treatment cohorts. Identification of poor preoperative prognostic factors is crucial for optimizing HCC treatment strategies.

Regarding liver factors and perioperative patient indicators, we found that older age, platelet count, lymphocyte count, albumin level, and CRP were significantly associated with a predicted mortality rate of ≥ 3%. Indicators related to these include nutritional and immune factors such as the PNI, GNRI, and Glasgow Prognostic Score (GPS). In a previous meta‐analysis, a low PNI, calculated from serum albumin and total lymphocyte count of < 45, was significantly associated with poor OS and RFS in patients with HCC who received hepatectomy [21]. Our previous study revealed that patients with a low PNI (cut‐off: 45) or low GNRI (cut‐off: 98) had significantly worse OS and RFS after initial hepatic resection [22]. Horino et al. [23] investigated the relationship between GPS and prognosis in 352 patients with HCC who underwent hepatectomy. According to the multivariate analysis, GPS (hazard ratio, 3.796; *p* < 0.001) was an independent poor prognostic factor, along with tumor size, operation time, and portal vein invasion. The CRP‐Albumin Lymphocyte index is a useful predictive biomarker for the postoperative prognosis of patients with HCC [24].

Numerous studies have reported that scoring systems that use serum albumin as a major component, such as the PNI, GNRI, and GPS, are significantly associated with outcomes in patients with HCC. Serum albumin functions as a multifaceted confounding factor that reflects not only nutritional status, but also hepatic synthesis capacity and systemic inflammatory status. The NCD risk calculator incorporates more comprehensive risk factors, including age, liver function, nutritional status, inflammatory markers, and comorbidities based on a nationwide database, making it highly suitable for assessing multilayered risks in patients with HCC. Therefore, the NCD risk calculator may function as an integrated “host vulnerability” index beyond existing individual biomarkers, potentially becoming a non‐invasive prognostic marker. Although this study suggested that host nutritional status, liver function, systemic inflammation, and immune responses may be involved in HCC prognosis, these mechanistic interpretations remain speculative. Therefore, careful interpretation of these results is necessary. Further research is required to investigate these mechanisms.

This study has several limitations. First, it is limited by its retrospective single‐center design. Second, there is a possibility of unobserved confounding and selection bias. The predicted mortality rate using the NCD risk calculator was calculated preoperatively, and patients who chose a non‐surgical treatment option based on this result were excluded from the study. Third, the surgery‐related mortality rate calculated using the NCD calculator may have overestimated the risk. The NCD calculator's risk model is based on data collected between 2011 and 2012. However, surgery‐related mortality from hepatic resection has declined in recent years [12]. Risk calculators may overestimate or underestimate results, and surgery‐related mortality, in particular, is likely to be overestimated because of the small number of cases. In fact, no surgery‐related mortality was observed in this study, which deviated from the predicted value. We must be mindful of the possibility of risk overestimating. However, this study highlights the usefulness of predicted mortality rate as a comprehensive tool for risk stratification. For patients with a predicted mortality rate of ≥ 3%, it was considered necessary to conduct thorough preoperative assessments, provide appropriate nutritional and rehabilitative interventions, and perform meticulous postoperative monitoring for recurrence.

In conclusion, a predicted mortality rate of ≥ 3% was an important predictor associated with poor long‐term prognosis, as well as short‐term postoperative outcomes in patients with HCC who underwent initial hepatic resection. These findings suggest that the NCD risk calculator may be a valuable complementary tool for decision‐making about liver resection in patients with HCC.

## Author Contributions

**Mariko Tsukagoshi:** conceptualization, writing – original draft, methodology, data curation, validation, formal analysis, investigation, visualization, project administration, writing – review and editing. **Kenichiro Araki:** conceptualization, investigation, validation, writing – review and editing, project administration. **Kouki Hoshino:** data curation, investigation. **Ryo Muranushi:** data curation, investigation. **Kei Hagiwara:** data curation, investigation. **Shunsuke Kawai:** data curation, investigation. **Ken Shirabe:** conceptualization, validation, writing – review and editing, supervision. **Takamichi Igarashi:** data curation, investigation. **Norio Kubo:** data curation, investigation.

## Funding

The authors have nothing to report.

## Ethics Statement

The study was approved by the Ethics Committee of the study hospital and was conducted in accordance with the institutional guidelines and the Declaration of Helsinki. Patient consent was obtained using the opt‐out method.

## Conflicts of Interest

The authors report no proprietary or commercial interest in any product mentioned or concept discussed in this article. Ken Shirabe is an editor‐in‐chief of the Annals of Gastroenterological Surgery. He was not involved in editorial handling, the selection of reviewers, or the final decision regarding this manuscript.

## Data Availability

The data that support the findings of this study are available from the corresponding author upon reasonable request.
